# The Integration of qSOFA with Clinical Variables and Serum Biomarkers Improves the Prognostic Value of qSOFA Alone in Patients with Suspected or Confirmed Sepsis at ED Admission

**DOI:** 10.3390/jcm9041205

**Published:** 2020-04-22

**Authors:** Filippo Mearelli, Giulia Barbati, Chiara Casarsa, Carlo Giansante, Andrea Breglia, Andrea Spica, Cristina Moras, Gaia Olivieri, Alessandro Agostino Occhipinti, Margherita De Nardo, Francesca Spagnol, Nicola Fiotti, Filippo Giorgio Di Girolamo, Maurizio Ruscio, Luigi Mario Castello, Efrem Colonetti, Rossella Marino, Claudio Ronco, Michela Zanetti, Enrico Lupia, Maria Lorenza Muiesan, Salvatore Di Somma, Gian Carlo Avanzi, Gianni Biolo

**Affiliations:** 1 Unit of Internal Medicine, Clinica Medica, Department of Medical Surgical and Health Sciences-University of Trieste, Strada di Fiume 447, 34100 Trieste, Italy; chiaracasarsa@gmail.com (C.C.); giansant@units.it (C.G.); enricofour@gmail.com (A.B.); spikejoe88@gmail.com (A.S.); cristinam8937@gmail.com (C.M.); olivieri.gaia@gmail.com (G.O.); alexander82@tiscali.it (A.A.O.); marghe.denardo@gmail.com (M.D.N.); francescaspagnol@yahoo.it (F.S.); fiotti@units.it (N.F.); fdigirolamo@gmail.com (F.G.D.G.); zanetti@units.it (M.Z.); biolo@units.it (G.B.); 2 Biostatistics Unit, Department of Medical Surgical and Health Sciences-University of Trieste, 34100 Trieste, Italy; gbarbati@units.it; 3 Department of Laboratory Medicine-University of Trieste, Strada di Fiume 447, 34100 Trieste, Italy; maurizio.ruscio@asuits.sanita.fvg.it; 4 Unit of Emergency Medicine, Department of Translational Medicine Eastern Piedmont-University of Novara, Corso Giuseppe Mazzini 18, 28100 Novara, Italy; castello@med.unipm.it (L.M.C.); avanzi@med.unipm.it (G.C.A.); 5 Unit of Internal Medicine, Department Of Clinical And Experimental Sciences-University Of Brescia, Via Marcantonio Ducco 44, 25100 Brescia, Italy; colonetti@gmail.com (E.C.); marialorenza.muiesan@unibs.it (M.L.M.); 6 Unit of Emergency Medicine, Department of Medical Surgery Sciences and Translational medicine-University “Sapienza” of Rome, Via di Grottarossa 1035/1039, 00189 Rome, Italy; rossellamarino1973@yahoo.it (R.M.); salvatore.disomma@uniroma1.it (S.D.S.); 7 Unit of Nephrology, Department of Nephrology, Dialysis and Transplantation International Renal Research Institute St Bortolo Hospital, Viale Ferdinando Rodolfi 37, 36100 Vicenza, Italy; cronco@goldnet.it; 8 Unit of Emergency Medicine, Department of Medical Sciences-University of Turin, Corso Bramante 88, 10126 Turin, Italy; enrico.lupia@unito.it

**Keywords:** sepsis, biomarkers, C-reactive protein, lactate, procalcitonin, mid-regional proadrenomedullin, soluble tumor necrosis factor receptor-1, soluble triggering receptor expressed on myeloid cell-1, presepsin, and soluble IL-2 receptor α

## Abstract

Background: The prognostic value of quick sepsis-related organ failure assessment (qSOFA) outside intensive care units has been criticized. Therefore, we aimed to improve its ability in predicting 30-day all-cause mortality, and in ruling out the cases at high risk of death among patients with suspected or confirmed sepsis at emergency department (ED) admission. Methods: This study is a secondary analysis of a prospective multicenter study. We built three predictive models combining qSOFA with the clinical variables and serum biomarkers that resulted in an independent association with 30-day mortality, in both 848 undifferentiated patients (Group 1) and in 545 patients definitively diagnosed with sepsis (Group 2). The models reaching the highest negative predictive value (NPV) with the minimum expenditure of biomarkers in Group 1 and in Group 2 were validated in two cohorts of patients initially held out due to missing data. Results: In terms of the area under the receiver-operating characteristic curve, all six models significantly exceeded qSOFA in predicting prognosis. An “extended” qSOFA (eqSOFA1) in Group 1 and an eqSOFA2 integrated with C-reactive protein and mid-regional proadrenomedullin (eqSOFA2+CRP+MR-proADM) in Group 2 reached the best NPV (0.94 and 0.93, respectively) and ease of use. eqSOFA1 and eqSOFA2+CRP+MR-proADM performed equally well in both the inception and validation cohorts. Conclusions: We have derived and validated two prognostic models that outweigh qSOFA in predicting mortality and in identifying the low risk of death among patients with suspected or confirmed sepsis at ED admission.

## 1. Introduction

Sepsis is a major cause of mortality worldwide. At emergency department (ED) admission, the proper assessment of sepsis outcome is vitally important to aid clinical decision making and to optimize the allocation of critical care resources. In 2016, the SEPSIS-3 Task Force proposed quick sepsis-related organ failure assessment (qSOFA), a simple and rapid score, to help discriminate sepsis survivors from non-survivors with a performance at least comparable with that of a sequential organ failure assessment (SOFA) score [1]. Several studies have demonstrated the usefulness of qSOFA-based prognostication in patients with suspected sepsis, especially when compared with that of the systemic inflammatory response syndrome (SIRS) criteria [2]. However, it was not always clearly stated in those works if patients were included who were initially suspected to have infection, but were adjudicated not to have infection on expert review. This finding, on one hand, makes those studies’ interpretation tricky, and on the other hand, it may raise concerns about their generalizability. Given the above premises, the prognostic value of qSOFA at ED admission has been questioned, especially when compared with more complex warning systems [2,3,4]. 

Six studies [5,6,7,8,9,10] sought to assess whether the integration of qSOFA with plasma concentrations of conventional biomarkers of prognosis (C-reactive protein [CRP] [5,6], lactate [7,8,9,10], and procalcitonin [5,6]) could improve the performance of qSOFA alone in predicting sepsis outcome: however, the results were conflicting. In the last 10 years, numerous circulating mediators of host response, such as mid-regional proadrenomedullin (MR-proADM ) [11], soluble tumor necrosis factor receptor-1 (sTNFR-1) [12,13,14,15,16,17], soluble triggering receptor expressed on myeloid cell-1 [18] (sTREM-1), presepsin [19], soluble phospholipase A2 group IIA (sPLA2GIIA) [20], and soluble IL-2 receptor α [21] (sIL2Rα) have been associated with poor prognosis in sepsis. Whether contemporary use of all of these experimental biomarkers could act synergistically with qSOFA in predicting outcome is unknown. Therefore, in this study, we tested the hypothesis that measuring concentrations of the aforementioned conventional and experimental biomarkers would enhance the prognostic value of qSOFA in undifferentiated patients with suspected sepsis at ED admission and in those, among them, definitively diagnosed with sepsis at the end of clinical work-up. 

## 2. Methods

This study is a secondary analysis of a prospective multicenter study conducted in 5 Italian EDs during a 2-year period [22]. Its original purpose was to derive and validate a predictive algorithm to rule out infection in 1132 undifferentiated patients with suspected sepsis who fulfilled at least 2 SIRS criteria at ED admission. The latter cohort included both patients who received a definitive diagnosis of infection and those who were deemed not infected (ni-SIRS) at the end of clinical work-up. Exclusion criteria were age less than 18 years, absence of informed consent, and pregnancy. Etiology, severity, and source of infection were adjudicated according to a predefined classification system (available in the Supplement). For this secondary analysis, we reviewed the three clinical variables required to calculate the qSOFA score, and the first venous lactate concentration measured at each respective site in all undifferentiated patients with suspected sepsis. The primary outcome of the present study was 30-day all-cause mortality. The secondary outcome was ruling out the high risk of death.

### 2.1. Biomarker Measurements

Blood samples for CRP, procalcitonin, sPLA2GIIA, presepsin, sIL2Rα, and sTREM-1 measurements were collected at ED admission in the entire cohort of undifferentiated patients with suspected sepsis, as previously reported [22]. MR-proADM and sTNFR-1 were measured specifically for this secondary analysis in the remaining frozen aliquots of serum. In detail, MR-proADM was assayed by an automated Kryptor analyzer (Kriptor Brahms AG, Hennigsdorg Germany) in the 1132 undifferentiated patients with suspected sepsis. Among these patients, in those who were definitively diagnosed with sepsis (729), sTNFR-1 determinations were carried out by commercial enzyme-linked immunosorbent assay kits following the manufacturer’s instructions (R&D). 

### 2.2. Statistical Analysis

Categorical data are summarized as percentages and numbers, normally distributed continuous data as mean ± standard deviation, and non-normally distributed variables as median and interquartile range. The unpaired Student *t* test was used, when appropriate, for the comparison of normally distributed data, while the Mann-Whitney test was used for non-normal continuous data. The χ2 test or the Fisher test was used, when appropriate, to compare categorical variables expressed as proportions. Unadjusted odds ratios (ORs) were calculated to assess the independent effect of qSOFA score, clinical variables, and serum biomarkers on 30-day mortality. 

To estimate a multivariable model, two strategies were implemented and compared: one based on a least absolute shrinkage and selection operator (LASSO) [23], i.e., a penalized logistic regression, and the other on a standard logistic full-model approach, in both cases starting from all variables significant at univariable analysis. For the lasso-based procedure, hyperparameter λ (penalty weight) were tuned with a default grid search implemented in the R “glmnet” library, in a 10-fold cross-validation. The same conclusions (in terms of the selected variables) were obtained with the two strategies. We therefore reported as results the odds ratios and corresponding 95% CI, estimated by the standard logistic regression model. We built three nested-predictive models, including the qSOFA score, the most significant clinical variables, and the biomarkers selected by the LASSO method. This was done both in undifferentiated patients with suspected sepsis at ED admission and in those, among them, definitively diagnosed with sepsis. 

Independent predictors were incrementally added to the qSOFA score starting from the easiest and cheapest to collect in clinical practice (i.e., from the clinical variables), through the conventional biomarkers and ending with the experimental biomarkers (unavailable for prime time). For the undifferentiated patients with suspected sepsis, the three predictive models were built as follows: Model 1 included the qSOFA score and clinical variables; Model 2 encompassed the qSOFA score, clinical variables, and conventional biomarkers; and Model 3 incorporated all of the above items and the experimental biomarkers. The same procedure was applied for the patients definitively diagnosed with sepsis (the models were termed Model 4, Model 5, and Model 6, respectively). Discrimination power was quantified using C statistics (area under the receiver-operating characteristic curve [AUROC]), and the De Long test was calculated to compare model AUROCs. According to the Youden Index method, different cut-offs were estimated between models on the estimated predicted probabilities of event, and compared in terms of sensitivity, specificity, positive predictive values (PPVs), negative predictive values (NPVs), and positive/negative likelihood ratios (LRs). The tests between NPVs were performed using the generalized score statistics [24]. All *p* values were two sided and considered significant when <0.05. All analyses were performed using the IBM SPSS statistic 24 software and the R software, R Core Team (2019), using the libraries “rms,” “Hmisc,”, “glmnet”, “OptimalCutPoints,” and “DTComPair”.

## 3. Results

### 3.1. Group 1: Undifferentiated Patients with Suspected Sepsis at ED Admission.

Of the 1132 undifferentiated patients with suspected sepsis, 304 were excluded because of missing data (Figure S1 available in the Supplement). We did not find a statistically significant difference between complete and incomplete cases except for median Charlson Index, sTREM-1, and presepsin concentrations (higher in complete than in noncomplete data, *p* = 0.01). Baseline characteristics of the 828 patients in Group 1 and their etiology of SIRS at the end of clinical work up are summarized in Table 1. 

**Figure 1:**
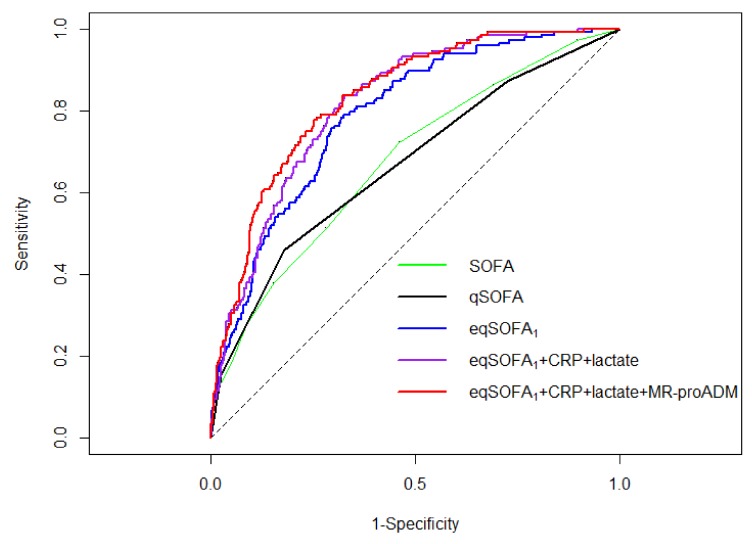
Group 1: Receiver operating characteristic curves of the sequential organ failure assessment (SOFA), quick sepsis-related organ failure assessment (qSOFA), “extended” qSOFA derived in Group 1 (eqSOFA_1_), eqSOFA_1_ integrated with serum concentrations of C-reactive protein and lactate (eqSOFA_1_+CRP+lactate) and eqSOFA_1_ combined with serum concentrations of C-reactive protein, lactate and mid–regional proadrenomedullin (eqSOFA_1_+CRP+lactate+MR-proADM) to predict 30-day mortality.

The 30-day all-cause mortality rate was 18%. In Group 1, the qSOFA and SOFA score showed a similar discriminatory power (AUROC for mortality 0.67; 95% CI 0.62–0.72 and 0.65; 95% CI 0.61–0.69, respectively; *p* = 0.7565). Independent predictors of outcome, which were selected to build the three predictive models, are shown in Table S1 (available in the Supplement). Model 1 represented an “extended” qSOFA (eqSOFA_1_): it encompassed gender, age, Charlson Index score, qSOFA score, body temperature <36 °C, heart rate >90/minute, and white blood cell count>12,000/mm^3^. Model 2 was based on the combination of eqSOFA_1_, CRP, and lactate (eqSOFA_1_+CRP+lactate). Model 3 included all of the latter and MR-proADM (eqSOFA_1_+CRP+lactate+MR-proADM). The receiver-operating characteristic (ROC) curves are available in Figure 1. 

Prognostic performance of the three predictive models is reported in Table 2. 

The AUROC of eqSOFA_1_ (0.79; 95% confidence interval [CI] 0.62–0.72), eqSOFA_1_+CRP+lactate (0.81; 95% CI 0.75–0.83), and eqSOFA_1_+CRP+lactate+MR-proADM (0.83; 95% CI 0.80–0.87) significantly exceeded that of qSOFA (all *p*<0.001). From the comparison of the three predictive models, two conclusions could be drawn: first, the AUROC for mortality of eqSOFA_1_+CRP+lactate was significantly better than that of eqSOFA_1_ (*p* = 0.01). Second, the addition of MR-proADM to eqSOFA_1_+CRP+lactate resulted in a further improvement of its predictive ability of 30-day mortality (*p* = 0.05). 

### 3.2. Group 2: Patients with Suspected Sepsis at ED Admission who Received a Definitive Diagnosis of Sepsis at the end of Clinical Work-Up. 

A total of 729 patients with suspected sepsis were definitively diagnosed with sepsis. There were 184 incomplete cases (Figure S1, available in the Supplement). Only the median concentration of sTREM-1 was significantly higher in patients with complete data than in those with incomplete data (*p* = 0.03). Table 3 summarizes the baseline characteristics of patients included in Group 2 (545), their etiology, and source of infection. 

A total of 122 patients died within 30 days from ED admission (23%). In Group 2, we did not find a statistically significant difference between the AUROC of the qSOFA and SOFA score in predicting sepsis outcome (0.66; 95% CI 0.61-0.71 and 0.65; 95% CI 0.59-0.69, respectively; *p* = 0.5639).

The same method of analysis was applied in Group 2 as in Group 1. The only difference was that, in Group 2, sTNFR-1 was added to the panel of biomarkers already tested in Group 1. This choice was elicited by the recent findings obtained in favor of using sTNFR-1 for sepsis prognostication in the intensive care unit (ICU) [17]. In accord with the final list of the independent predictors of 30-day mortality (Table S2, available in the Supplement), the three predictive models were built as follows: Model 4 included gender, age, Charlson Index, qSOFA, body temperature < 36°C, and heart rate >90/min (eqSOFA_2_); Model 5 added CRP to eqSOFA_2_ (eqSOFA_2_+CPR); and Model 6 enclosed eqSOFA_2_, CRP, and MR-proADM (eqSOFA_2_+CRP+MR-proADM). Sensitivity, specificity, NPV, PPV, LR-, and LR+ of the three models are reported in Table 4. 

**Figure 2:**
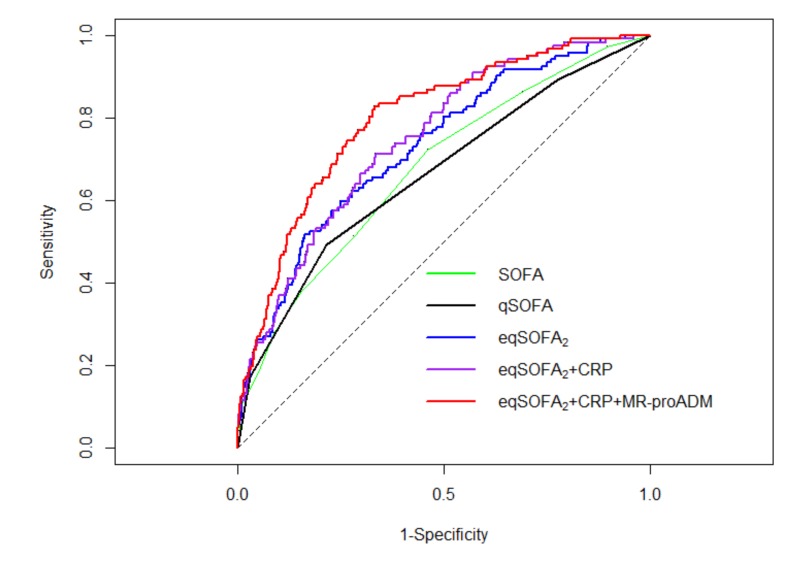
Group 2: Receiver operating characteristic curves of the sequential organ failure assessment (SOFA), quick sepsis-related organ failure assessment (qSOFA), “extended” qSOFA_2_ derived in Group 2 (eqSOFA_2_), eqSOFA_2_ integrated with serum concentrations of C-reactive protein (eqSOFA_2_+CRP) and eqSOFA_2_ combined with serum concentrations of C-reactive protein and mid–regional proadrenomedullin (eqSOFA_2_+CRP+MR-proADM) to predict 30-day mortality.

Their ROC curves are shown in Figure 2. 

eqSOFA_2_ (AUROC 0.73; 95% CI 0.68–0.78), eqSOFA_2_+CRP (AUROC 0.75; 95% CI 0.70–0.79), and eqSOFA_2_+CRP+MR-proADM (AUROC 0.80; 95% CI 0.76–0.84) proved to be significantly better than qSOFA (AUROC 0.66; 95% CI 0.61–0.71; all *p* < 0.001) in predicting outcome. We did not observe a significant difference between the AUROC for mortality of eqSOFA_2_ and eqSOFA_2_+CRP (*p* = 0.068). On the contrary, the prognostic performance of eqSOFA_2_+CRP was significantly improved by its integration with MR-proADM (*p* = 0.002). 

## 4. Discussion

Patients initially suspected to have an infection, but who were deemed ni-SIRS on expert review, were excluded from the first prospective study, which was conducted to validate the prognostic accuracy of qSOFA in patients with suspected sepsis at ED admission [25]. In our study, 10% of these patients had an ni-SIRS result at the end of clinical work-up. These epidemiological findings resembled that of previous works performed in the same setting [26,27], and reinforced the need to evaluate the prognostic performance of qSOFA in a population that mirrors “real life” patients with suspected sepsis at ED admission. Therefore, we assessed the predictivity of qSOFA not only in patients definitively diagnosed with sepsis (Group 2), but also in undifferentiated patients with suspected sepsis (Group 1). Interestingly, the prognostic accuracy shown by qSOFA in Group 1 and in Group 2 was similarly far from perfection (AUROC 0.67 and 0.66, respectively). To corroborate this finding, we have to highlight that an AUROC for mortality under 0.7 was also reached by qSOFA in the largest prospective study ever performed to assess its prognostic accuracy in patients with suspected sepsis at ED admission^3^. 

As stated in the method section, the first step to enhance the predictivity of qSOFA entailed its combination with the clinical variables independently associated with the outcome in each cohort. In Group 1, this meant building a predictive model (eqSOFA_1_) based on gender, age, Charlson Index score, qSOFA score, and three of the SIRS criteria (body temperature <36°C, heart rate >90/minute, and white blood cell count>12,000/mm^3^). The same clinical variables, except for white blood cell count >12,000/mm^3^, composed the eqSOFA_2_ in Group 2. It follows that, eqSOFA is not a new warning score, but rather the synthesis, adjusted for age, gender, and comorbidities, of two existing ones. For the first time in the literature, the SIRS criteria and qSOFA were proven to act synergistically to predict outcome in patients with suspected sepsis at ED admission; furthermore, studies do not exist that have derived a biomarker-enriched model predictive of prognosis specifically for undifferentiated patients with suspected sepsis (eqSOFA_1_) and for those, among them, definitively diagnosed with sepsis (eqSOFA_2_). Notably, the prognostic performance of both eqSOFA_1_ and eqSOFA_2_ exceeded that of qSOFA (AUROC 0.79 and 0.73, respectively; all *p* < 0.001). When eqSOFA_1_ and eqSOFA_2_ were enriched with the biomarkers that were proved to be independently associated with the outcome in each group, their predictive ability of 30-day mortality was further enhanced. Of the six prognostic models derived in this study, eqSOFA_1_+CRP+lactate+MR-proADM in Group 1, and eqSOFA_2_+CRP+MR-proADM in Group 2 exhibited the highest AUROC for mortality with the maximum expenditure of biomarkers (0.83 and 0.80, respectively). A similar performance was reached by Mikacenic et al in the largest study ever conducted to derive and validate a biomarker-enriched model to predict sepsis outcome in the ICU (AUROC 0.79) [16]. Among all of the putative biomarkers, only IL-8 and sTNFR-1 were included in that “robust” model. We may speculate that if the prognostic performance of eqSOFA_1_+CRP+lactate+MR-proADM and eqSOFA_2_+CRP+MR-proADM was to be confirmed in the ICU setting, the key issue in favor of using our models would be that both of these model could be promptly implemented in clinical practice with CRP, lactate, and MR-proADM, unless IL-8 and sTNFR-1, are already available for prime time.

Seeing as the introduction of qSOFA, as compared with SIRS-based prognostication, has enhanced the specificity of the prediction of mortality associated with sepsis, but at the expense of sensitivity^2^, our analysis focused on predictive models that exhibited the highest NPV, possibly, with the minimum expenditure of biomarkers. The main goal of such an approach was to identify the models with the best ability to rule out the outcome, while easing the financial burden of the use of biomarkers in the health care system. The proper identification of subjects at low risk of death is particularly pressing in Europe, where, as in other countries with poor availability of ICU beds, most of the patients with suspected sepsis are initially managed in the ED and then transferred to general wards [28]. It would be desirable to identify a screening tool that could support ED physicians in the safe admission of patients with suspected sepsis outside the ICU, to avoid the risk of a less than optimum resuscitation. Unfortunately, qSOFA did not meet this need in either Group 1 (NPV = 0.84; 95% CI 0.82-0.90) or Group 2 (NPV = 0.80; 95% CI 0.79-0.87). Both eqSOFA_1_ and eqSOFA_2_ outperformed qSOFA in ruling out poor prognosis (all *p* < 0.001). However, depending on the etiology of SIRS, eqSOFA could be employed as a “stand alone” model for the safe identification of subjects at low risk of death among patients with suspected sepsis at ED admission. In fact, while the NPV of eqSOFA_1_ was sufficiently high to support its use for a safe ruling out of patients at high risk of mortality (0.94; 95% CI 0.91-0.97), the NPV eqSOFA_2_ was too low for this purpose (0.85; 95% CI 0.81-0.89). The contribution of biomarkers to further improve the NPV of eqSOFA was not straightforward in either group. In fact, the ability of eqSOFA_1_ to rule out poor prognosis was not ameliorated by its enrichment with CRP, lactate, and MR-proADM (0.94), whereas CRP, and MR-proADM significantly increased the NPV of eqSOFA_2_ to 0.93 (*p* < 0.001). Outstandingly, eqSOFA_1_ and eqSOFA_2_+CRP+MR-proADM obtained the highest NPV to identify the patients at low risk of death, using as few biomarkers as possible, and without the need to maximize their threshold score [17]. We want to emphasize that eqSOFA_1_ in Group 1 and eqSOFA_2_+CRP+MR-prodADM in Group 2 reached such a performance without worsening the corresponding PPV of qSOFA (Group 1 = 0.36 and 0.40, respectively; Group 2 = 0.40 and 0.42, respectively). 

These findings can be translated to clinical practice as follows: eqSOFA_1_ could be particularly helpful for recognizing subjects at low risk of death among patients with suspected sepsis at ED admission, for whom it is often challenging to pose a definitive diagnosis of sepsis, because the early presentation of many acute pathologies closely mimics that of sepsis, and the results of the microbiological work-up are usually not available. When the diagnosis of sepsis is already established (which occurs mainly in general wards), eqSOFA_2_+CRP+MR-proADM should be used in place of eqSOFA_1_, to rule out patients at high risk of mortality with the same high predictivity exhibited by eqSOFA_1_ in undifferentiated patients.

This is the largest sample to date of patients with suspected sepsis at ED admission, who had all eight biomarkers measured. It is worth nothing that, for the first time in the literature, this panel of conventional and experimental biomarkers (including two of the most promising, MR-proADM and sTNFR-1) was compared side by side with qSOFA in the same study for sepsis prognostication. In the light of these premises, some interesting conclusions can be drawn. First, MR-proADM, as standalone biomarker to predict outcome, exhibited an AUROC higher than that of both qSOFA and all the other biomarkers independently from etiology of SIRS (Table S3 and Table S4). Second, among all the biomarkers assayed in our study, only CRP and MR-proADM were independently associated with 30-day mortality in either Group 1 or Group 2. Third, CRP and MR-prodADM proved to be addictive to eqSOFA in predicting prognosis in both cohorts. Fourth, lactate resulted an independent biomarker of prognosis only in Group 1: it contributed with eqSOFA_1_, CRP and MR-proADM to build the model with the highest AUROC to predict mortality (qSOFA_1_+CRP+lactate+MR-proADM). Fifth, biomarkers were useless in improving the NPV of eqSOFA_1_; however, in Group 2, CRP and MR-proADM were necessary to significantly increase the ability of eqSOFA_2_ in ruling out adverse outcome. 

The study does have limitations. A total of 304 undifferentiated patients with suspected sepsis did not enter the final analysis. To mitigate the concerns related to the generalizability of eqSOFA_1_ and eqSOFA+CRP+MR-proADM_2_, we validated our models in two cohorts of patients initially held out due to missing data. The details are discussed in the Supplement. In summary, in terms of AUROC, the prognostic performance of eqSOFA_1_ and eqSOFA_2_+CRP+MR-proADM in the inception (0.79 and 0.80 [Figure S2], respectively) and validation cohorts (0.80 and 0.82 [Figure S3], respectively) was similar (*p* = 0.82, *p* = 0.81, respectively). Moreover, we did not assess the correlation of eqSOFA_1_ and eqSOFA_2_+CRP+MR-proADM with the length of hospital stay, disease progression, or requirement for ICU care after ED admission. 

In conclusion, we have derived and validated two prognostic models that were proven to be more robust than qSOFA and SOFA in predicting mortality and in ruling out the high risk of death among patients with suspected sepsis at ED admission, either undifferentiated (eqSOFA_1_) or definitively diagnosed with sepsis (eqSOFA_2_+CRP+MR-proADM). The two predictive models, which were composed of clinical variables routinely measured at ED admission, and encompassed biomarkers that are already available for prime time, need to be externally validated prior to any clinical application. 

## Figures and Tables

**Table 1:** Group 1: baseline patient characteristics and etiology of systemic inflammatory response syndrome (SIRS) at the end of the clinical work-up.

	Survivor *n* = 680	Non Survivor *n* = 148	*p*	OR
Male	368	67	0.051	1.426 (0.998–2.038)
Median age (IQR)	78 (69–86)	85 (79–90)	<0.001	1.051 (1.032–1.070)
Median Charlson Index (IQR)	2 (1–4)	3 (2–5)	<0.001	1.164 (1.085–1.250)
Medain SOFA (IQR)	2 (1–4)	4 (2–6)	<0.001	1.337 (1.238–1.444)
Median qSOFA (IQR)	1 (0–1)	1 (1–2)	<0.001	2.419 (1.919–3.050)
Clinical variables				
Body temperature				
>38°C	301 (44)	41 (28)	<0.001	
<36°C	12 (2)	18 (12)	<0.001	7.708 (3.636–16.386)
White blood cell count				
>12,000/mm^3^	370 (54)	101 (68)	0.002	1.800 (1.234–2.626)
<4000/mm^3^	30 (4)	4 (3)	0.342	
RR > 20/min or pCO_2_ < 32 mmHg	508 (75)	128 (86)	0.002	2.167 (1.312–3.580)
Heart rate > 90/min	509 (75)	122 (82)	0.05	1.576 (0.998–2.491)
Median BM (IQR)^				
C–reactive protein (mg/dL)	73 (20–160)	117 (52–205)	<0.001	1.441 (1.231–1.687)
Lactate (mg/dL)	13 (9–19)	17 (12–26)	<0.001	2.491 (1.788–3.471)
Procalcitonin (ng/mL)	0.33 (0.13–2.22)	0.9 (0.24–3.7)	<0.001	1.178 (1.025–1.354)
sIL2Rα (pg/mL)	13234 (8026–22158)	20028 (12555–3269	<0.001	1.908 (1.508–2.414)
sTREM–1 (pg/mL)	375 (260–590)	633 (409–1001)	<0.001	3.102 (2.332–4.126)
sPLA_2_GIIA (ng/mL)	31 (21–35)	31 (26–35)	0.041	1.638 (1.149–2.334)
Presepsin (pg/mL)	443 (280–908)	802(476–1450)	<0.001	1.746 (1.446–2.107)
MR–proADM (nmol/L)	1.6 (1.1–2.5)	2.8 (1.9–4.7)	<0.001	4.361 (3.033–6.269)
Etiology of SIRS				
ni-SIRS	81(93)	6 (7)	0.05	
Infections				
Localized infections	95 (99)	1 (1)	<0.001	
Sepsis	400 (81)	96 (19)	0.096	
Septic shock	23 (47)	26 (53)	<0.001	
d-SIRS	81 (81)	19 (19)	0.754	

Abbreviations: OR = odds ratio, IQR = interquartile range, SOFA = sequential organ failure assessment, Qsofa = quick sepsis-related organ failure assessment, RR = respiratory rate, ni-SIRS = non infective systemic inflammatory response syndrome, d-SIRS = debatable systemic inflammatory response syndrome, sIL2Rα = soluble IL-2 receptor α, sTREM-1 = soluble triggering receptor expressed on myeloid cell-1, sPLA_2_GIIA = soluble phospholipase A_2_ group IIA, and MR-proADM = mid-regional proadrenomedullin. ^ Data are n° (%) unless otherwise specified.

**Table 2:** Group 1: performance of the quick sepsis-related organ failure assessment (qSOFA), Model 1, Model 2, and Model 3.

	AUROC	Sensitivity	Specificity	NPV	PPV	LR-	LR+
SOFA	0.65	0.70	0.53	0.83	0.25	0.51	1.57
(cut-off 3)	(0.61–0.69)	(0.61–0.78)	(0.50–0.58)	(0.81–0.91)	(0.23–0.33)	(0.39–0.67)	(1.36–1.79)
qSOFA	0.67	0.46	0.82	0.84	0.36	0.66	2.58
(cut-off 2)	(0.62–0.72)	(0.38–0.54)	(0.79–0.85)	(0.82–0.90)	(0.32–0.44)	(0.56–0.77)	(2.03–3.28)
eqSOFA_1_^a^	0.79	0.79	0.68	0.94	0.35	0.31	2.43
(cut-off* 0.16)	(0.75–0.83)	(0.72–0.86)	(0.63–0.71)	(0.91–0.97)	(0.31–0.45)	(0.23-0.43)	(2.12–2.79)
eqSOFA_1_+CRP+lactate^b^	0.81	0.81	0.67	0.94	0.36	0.24	2.55
(cut-off* 0.15)	(0.78–0.85)	(0.77–0.89)	(0.64–0.71)	(0.90–0.96)	(0.32–0.47)	(0.17–0.35)	(2.25–2.91)
eqSOFA_1_+CRP+lactate+MR-proADM^c^	0.83	0.82	0.75	0.94	0.40	0.30	3.09
(cut-off* 0.18)	(0.80–0.87)	(0.70–0.84)	(0.71–0.78)	(0.92–0.96)	(0.36–0.51)	(0.22–0.40)	(2.64–3.61)

Abbreviations: SOFA= sequential organ failure assessment, eqSOFA_1_= “extended” quick sepsis-related organ failure assessment derived in Group 1, CRP= C-reactive protein, MR-proADM= mid-regional proadrenomedullin, AUROC= area under the receiver operating characteristic curve, NPV=negative predictive value, PPV=positive predictive value, LR-=negative likelihood ratio, and LR+=positive likelihood ratio. ()=95% Confidence Interval. ^a^Model 1: gender, age, Charlson Index score, qSOFA score, body temperature <36°C, heart rate >90/minute, and white blood cell>12,000/mm^3^. ^b^Model 2: gender, age, Charlson Index score, qSOFA score, body temperature <36°C, heart rate >90/minute, white blood cell>12,000/mm^3^, Log C-reactive protein, and Log lactate. ^c^Model 3: gender, age, Charlson Index score, qSOFA score, body temperature <36°C, heart rate >90/minute, white blood cell>12,000/mm^3^, Log C-reactive protein, Log lactate, and Log mid-regional proadrenomedullin *Cut-off of the models were selected according to the Youden Index method.

**Table 3:** Group 2: baseline patient characteristics, etiology, and source of infection at the end of the clinical work-up.

	Survivor *n* = 423 (%)	Non Survivor *n* = 122 (%)	*p*	OR
Male	242 (42)	56 (53)	0.027	0.635 (0.423–0.951)
Median age	80 (73–86)	85 (79–90)	<0.001	1.043 (1.021–1.065)
Median Charlson index	3 (1–4)	3 (2–5)	0.142	1.072 (0.984–1.169)
Median SOFA (IQR)	3 (2–4)	4 (3–6)	<0.001	1.27 (1.16–1.38)
Median qSOFA	1 (1–1)	2 (1–2)	<0.001	2.776 (1.838–4.193)
**Clinical variable**			
Body Temperature				
>38°C	204 (52)	39 (31)	0.001	
<36°C	9 (2)	16 (13)	<0.001	6.943 (2.985–16.149)
White blood cell count				
>12,000/mm^3^	241 (60)	82 (65)	0.043	1.800 (1234–2626)
<4000/mm^3^	18 (4)	4 (4)	0.629	
Heart rate>90 bpm	311 (73)	109 (80)	0.095	2.167 (1.312–3.580)
RR>20/min or pCO_2_<32 mmHg	328 (x)	108 (x)	0.008	2.234 (1.224–4.078)
**Median biomarker (IQR)**				
C-reactive protein (mg/dl)	129 (73-209)	130 (74-209)	0.039	1.369 (1.123–1.644)
Lactate (mg/dl)	14.4 (9.9-20)	18.3 (12.2-27.2)	0.007	1.863 (1.296–2.680)
Procalcitonin (ng/mL)	1.14 (0.36–5.09)	0.74 (0.26–5.47)	0.061	1.087 (0.934–1.266)
MR-proADM (nmol/L)	1.9 (1.3–3.2)	3.02 (1.96–5.04)	<0.001	3.411 (2.275–5.113)
sIL2Rα (pg/ml)	15130 (9741–25220)	19446 (12596–31520)	0.014	1.630 (1.246–2.133)
Presepsin (pg/mL)	546 (341–1047)	781 (446–1423)	0.023	1.545 (1.239–1.926)
sTREM-1 (pg/mL)	418 (280–711)	619 (405–989)	<0.001	2.359 (1.727–3.223)
sPLA_2_GIIA (ng/mL)	32.3 (27.4–36.3)	32.2 (28.8–36.5)	0.692	1.187 (0.768–1.834)
sTNFR-1 (pg/ml)	423.5 (320.4–634.0)	560.61 (388.2–712.6)	<0.001	3.164 (1.946–5.143)
**Source of infection**				
Single source	399 (94)	98 (80)	0.001	
LRTI	240 (56)	71 (58)	0.024	
Non LRTI	159 (38)	27 (22)	0.092	
Multiple source	24 (6)	24 (20)	<0.001	
**Etiology of infection**				
Clinically documented	261 (61)	80 (66)	0.345	
Microbiologically documented	174 (41)	51 (41)	0.932	
Monomicrobial	144 (34)	39 (31)	0.669	
Bacterial	133 (31)	41 (34)	0.814	
Gram positive	44 (10)	19 (15)	0.002	
Gram negative	77 (18)	11 (15)	0.037	
Non bacterial	10 (2)	1 (0)	0.307	
Polymicrobial	30 (7)	12 (1)	0.317	
BSI	70 (16)	29 (24)	0.365	
Non BSI	106 (25)	25 (20)	0.298	

Abbreviations: OR= odds ratio, IQR= interquartile range, SOFA= sequential organ failure assessment, qSOFA=quick sepsis-related organ failure assessment, RR=respiratory rate, LRTI=lower respiratory tract infections, BSI=blood stream infections, MR-proADM=mid-regional proadrenomedullin, sIL2Rα=soluble IL-2 receptor α, sTREM-1=soluble triggering receptor expressed on myeloid cell-1, sPLA_2_GIIA= soluble phospholipase A_2_ group IIA, and sTNFR-1-= soluble tumor necrosis factor receptor-1 (sTNFR-1). Data are n° (%) unless otherwise specified.

**Table 4:** Group 2: performance of the quick sepsis-related organ failure assessment (qSOFA), Model 4, Model 5, and Model 6.

	AUROC	Sensitivity	Specificity	NPV	PPV	LR-	LR+
SOFA	0.65	0.75	0.45	0.83	0.28	0.52	1.39
(cut-off 3)	(0.59–0.69)	(0.67–0.80)	(0.40–0.50)	(0.81–0.88)	(0.24–0.38)	(0.37–0.70)	(1.21–1.59)
qSOFA	0.66	0.49	0.78	0.80	0.40	0.65	2.29
(cut-off 2)	(0.61–0.71)	(0.40–0.58)	(0.74–0.82)	(0.79–0.87)	(0.34–0.49)	(0.54–0.77)	(1.77–2.95)
eqSOFA_2_^a^	0.73	0.52	0.84	0.85	0.48	0.58	3.21
(cut-off* 0.30)	(0.68–0.78)	(0.42–0.61)	(0.80–0.87)	(0.81–0.89)	(0.42–0.57)	(0.48–0.70)	(2.43–4.24)
eqSOFA_2_+CRP^b^	0.75	0.71	0.67	0.88	0.38	0.43	2.14
(cut-off* 0.22)	(0.70–0.79)	(0.62–0.79)	(0.62–0.71)	(0.84–0.91)	(0.33–0.48)	(0.32–0.57)	(1.79–2.55)
eqSOFA_2_+CRP+MR-proADM^c^	0.80	0.83	0.67	0.93	0.42	0.26	2.50
(cut-off* 0.18)	(0.76–0.84)	(0.75–0.89	(0.62–0.71)	(0.89–0.94)	(0.37–0.55)	(0.17–0.38)	(2.14–2.93)

Abbreviations: SOFA= sequential organ failure assessment, qSOFA= quick sepsis-related organ failure assessment, eqSOFA_2_= “extended” quick sepsis-related organ failure assessment derived in Group 2, CRP= C reactive protein, MR-proADM= mid-regional proadrenomedullin, AUROC= area under the receiver operating characteristic curve, NPV=negative predictive value, PPV=positive predictive value, LR-=negative likelihood ratio, LR+=positive likelihood ratio. ()=95% Confidence Interval. ^a^Model 4: gender, age, Charlson Index score, qSOFA score, body temperature < 36°C, and heart rate >90/min. ^b^Model 5: gender, age, Charlson Index score, qSOFA score, body temperature < 36°C, heart rate >90/min, and Log C-reactive protein. ^c^Model 6: gender, age, Charlson Index score, qSOFA score, body temperature < 36°C, heart rate >90/min, Log C-reactive protein, and Log MR-proADM. * Cut-off of the models were selected according to the Youden Index method.

## Supplementary Materials

The following are available online at https://www.mdpi.com/2077-0383/9/4/1205/s1, Figure S1. Flow chart describing the design of the inception (Group 1 and Group 2) and validation cohorts. Figure S2. Receiver operating characteristic curve of “extended” quick sepsis-related organ failure assessment derived in Group 1 (eqSOFA1) to predict 30-day mortality in the validation cohort. Figure S3. Receiver operating characteristic curve of the “extended” quick sepsis-related organ failure assessment derived in Group 2 integrated with serum concentrations of C-reactive protein and mid –regional proadrenomedullin (eqSOFA2+CRP+MR-proADM) to predict 30-day mortality in the validation cohort. Table S1. Independent predictors of 30-day mortality in Group 1: multivariable analysis. Table S2. Independent predictors of 30-day mortality in Group 2: multivariable analysis. Table S3. Prognostic performance of the biomarkers assayed in Group 1. Table S4. Prognostic performance of the biomarkers assayed in Group 2.

## References

[1] Singer M., Deutschman C.S., Seymour C.W., Shankar-Hari M., Annane D., Bauer M., Bellomo R., Bernard G.R., Chiche J.-D., Coopersmith C.M. (2016). The Third International Consensus Definitions for Sepsis and Septic Shock (Sepsis-3). JAMA.

[2] Serafim R., Gomes J.A., Salluh J., Póvoa P. (2018). A Comparison of the Quick-SOFA and Systemic Inflammatory Response Syndrome Criteria for the Diagnosis of Sepsis and Prediction of Mortality: A Systematic Review and Meta-Analysis. Chest.

[3] Churpek M., Snyder A., Han X., Sokol S., Pettit N., Howell M.D., Edelson D.P. (2017). Quick Sepsis-related Organ Failure Assessment, Systemic Inflammatory Response Syndrome, and Early Warning Scores for Detecting Clinical Deterioration in Infected Patients outside the Intensive Care Unit. Am. J. Respir. Crit. Care Med..

[4] Moskowitz A., Patel P.V., Grossestreuer A.V., Chase M., Shapiro N.I., Berg K., Cocchi M.N., Holmberg M.J., Donnino M.W. (2017). Quick Sequential Organ Failure Assessment and Systemic Inflammatory Response Syndrome Criteria as Predictors of Critical Care Intervention Among Patients With Suspected Infection. Crit. Care Med..

[5] Yu H., Nie L., Liu A., Wu K., Hsein Y.-C., Yen D.W., Lee M.-T.G., Lee C.-C. (2019). Combining procalcitonin with the qSOFA and sepsis mortality prediction. Med. Baltimore.

[6] Saeed K., Wilson D.C., Bloos F., Schuetz P., van der Does Y., Melander O., Hausfater P., Legramante J.M., Claessens Y.E., Amin D. (2019). The early identification of disease progression in patients with suspected infection presenting to the emergency department: A multi-centre derivation and validation study. Crit Care.

[7] Henning D.J., Puskarich M.A., Self W.H., Howell M.D., Donnino M.W., Yealy N.M., Jones A.E., Shapiro N.I. (2017). An Emergency Department Validation of the SEP-3 Sepsis and Septic Shock Definitions and Comparison with 1992 Consensus Definitions. Ann. Emerg. Med..

[8] Ho K.M., Lan N.S. (2017). Combining quick Sequential Organ Failure Assessment with plasma lactate concentration is comparable to standard Sequential Organ Failure Assessment score in predicting mortality of patients with and without suspected infection. J. Crit. Care.

[9] Baumann B.M., Greenwood J.C., Lewis K., Nuckton T.J., Darger B., Shofer F., Troeger D., Jung S.Y., Kilgannon J.H., Rodriguez R.M. (2019). Combining qSOFA criteria with initial lactate levels: Improved screening of septic patients for critical illness. Am. J. Emerg. Med..

[10] Seymour C.W., Liu V.X., Iwashyna T.J., Brunkhorst F.M., Rea T.D., Scherag A., Rubenfeld G., Kahn J.M., Shankar-Hari M., Singer M. (2016). Assessment of Clinical Criteria for Sepsis: For the Third International Consensus Definitions for Sepsis and Septic Shock (Sepsis-3). JAMA.

[11] Onal U., Valenzuela-Sánchez F., Vandana K., Rello J. (2018). Mid-Regional Pro-Adrenomedullin (MR-proADM) as a Biomarker for Sepsis and Septic Shock: Narrative Review. Healthcare.

[12] Mat-Nor M.B., Ralib A.M., Abdulah N.Z., Pickering J.W. (2016). The diagnostic ability of procalcitonin and interleukin-6 to differentiate infectious from noninfectious systemic inflammatory response syndrome and to predict mortality. J. Crit. Care.

[13] Wong H.R., Lindsell C.J., Pettilä V., Meyer N.J., Thair S.A., Karlsson S., Russell J.A., Fjell C., Boyd J.H., Ruokonen E. (2014). A multibiomarker-based outcome risk stratification model for adult septic shock. Crit. Care Med..

[14] De Pablo R., Monserrat J., Reyes E., Diaz-Martin D., Zapata M.R., Carballo F., De La Hera A., Prieto A., Alvarez-Mon M. (2011). Mortality in patients with septic shock correlates with anti-inflammatory but not proinflammatory immunomodulatory molecules. J. Intensiv. Care Med..

[15] Ware L.B., Koyama T., Zhao Z., Janz D.R., Wickersham N.E., Bernard G.R., May A.K., Calfee C.S., Matthay M.A. (2013). Biomarkers of lung epithelial injury and inflammation distinguish severe sepsis patients with acute respiratory distress syndrome. Crit. Care.

[16] Wong H.R., Cvijanovich N.Z., Anas N., Allen G.L., Thomas N.J., Bigham M.T., Weiss S.L., Fitzgerald J.C., Checchia P.A., Meyer K. (2015). A Multibiomarker-Based Model for Estimating the Risk of Septic Acute Kidney Injury. Crit. Care Med..

[17] Mikacenic C., Price B.L., Harju-Baker S., O’Mahony D.S., Robinson-Cohen C., Radella F., Hahn W.O., Katz R., Christiani D.C., Himmelfarb J. (2017). A Two-Biomarker Model Predicts Mortality in the Critically Ill with Sepsis. Am. J. Respir. Crit. Care Med..

[18] Su L., Liu D., Chai W., Liu D., Long Y. (2016). Role of sTREM-1 in predicting mortality of infection: A systematic review and meta-analysis. BMJ Open.

[19] Yang H.S., Hur M., Yi A., Kim H., Lee S., Kim S.-N. (2018). Prognostic value of presepsin in adult patients with sepsis: Systematic review and meta-analysis. PLoS ONE.

[20] Zeiher B.G., Steingrub J., Laterre P.F., Dmitrienko A., Fukiishi Y., Abraham E., EZZI Study Group (2005). LY315920NA/S-5920, a selective inhibitor of group IIA secretory phospholipase A2, fails to improve clinical outcome for patients with severe sepsis. Crit. Care Med..

[21] Matera G., Puccio R., Giancotti A., Quirino A., Pulicari M.C., Zicca E., Caroleo S., Renzulli A., Liberto M.C., Focà A. (2013). Impact of interleukin-10, soluble CD25 and interferon-γ on the prognosis and early diagnosis of bacteremic systemic inflammatory response syndrome: A prospective observational study. Crit. Care.

[22] Mearelli F., Fiotti N., Giansante C., Casarsa C., Orso D., De Helmersen M., Altamura N., Ruscio M., Castello L.M., Colonetti E. (2018). Derivation and Validation of a Biomarker-Based Clinical Algorithm to Rule Out Sepsis From Noninfectious Systemic Inflammatory Response Syndrome at Emergency Department Admission: A Multicenter Prospective Study. Crit. Care Med..

[23] Tibshirani R. (1996). Regression shrinkage and selection via the lasso. J. R. Stat. Soc. Ser. B.

[24] Leisenring W., Alono T., Pepe M.S. (2000). Comparisons of predictive values of binary medical diagnostic tests for paired designs. Biometrics.

[25] Freund Y., Lemachatti N., Krastinova E., Van Laer M., Claessens Y.-E., Avondo A., Occelli C., Feral-Pierssens A.-L., Truchot J., Ortega M. (2017). Prognostic Accuracy of Sepsis-3 Criteria for In-Hospital Mortality Among Patients With Suspected Infection Presenting to the Emergency Department. JAMA.

[26] Llewelyn M.J., Berger M., Gregory M., Ramaiah R., Taylor A.L., Curdt I., Lajaunias F., Graf R., Blincko S.J., Drage S. (2013). Sepsis biomarkers in unselected patients on admission to intensive or high-dependency care. Crit. Care.

[27] Heffner A., Horton J.M., Marchick M.R., Jones A.E. (2010). Etiology of illness in patients with severe sepsis admitted to the hospital from the emergency department. Clin. Infect. Dis..

[28] Levy M.M., Artigas A., Phillips G.S., Rhodes A., Beale R., Osborn T., Vincent J.-L., Townsend S., Lemeshow S., Dellinger R.P. (2012). Outcomes of the Surviving Sepsis Campaign in intensive care units in the USA and Europe: A prospective cohort study. Lancet Infect. Dis..

