# Supplementary material for: The Integration of qSOFA with Clinical Variables and Serum Biomarkers Improves the Prognostic Value of qSOFA Alone in Patients with Suspected or Confirmed Sepsis at ED Admission
Source: J Clin Med. 2020 Apr 22;9(4):1205. doi: 10.3390/jcm9041205 (PMC7230329; doi:10.3390/jcm9041205)
Supplement: Supplementary file 1 [file jcm-09-01205-s001.pdf]

**SUPPLEMENT**

**PREDEFINED CLASSIFICATION SYSTEM (PDCS)**

The PDCS was based on guidelines established by the main Authorities in the field of infection[1-7] and was approved, before the start of enrolment, after discussion among principal investigators.

*Comorbidities*

The following comorbidities were collected in this study: prior myocardial infarction (history not solely ECG changes), congestive heart failure, peripheral vascular disease (included aortic aneurysm ≥ 6 cm), chronic dementia (decline in at least 2 brain functions), chronic respiratory disease (include chronic obstructive pulmonary disease [COPD]), structural lung diseases such as bronchiectasis and interstitial lung disease), connective tissue diseases/vasculitis, peptic ulcer, liver disease, diabetes mellitus, chronic renal disease (defined as abnormal basal creatinine), solid cancer (was adjudicated if active at the time of presentation or requiring antineoplastic treatment within the previous five years), hematologic cancer, HIV and related syndromes.

*Etiology of infection*

Clinically documented infections comprised cases with a clinical history and a course suggestive of infection without supporting gram stain or cultures. Microbiologically documented infections included cases with a microbiological confirmation of infection. The latter were further subdivided into monomicrobial and polymicrobial infections according to the number of pathogens identified in the biological samples. Monomicrobial infections were defined as infections caused by a single germ: in this group, the bacterial and nonbacterial infections were adjudicated depending on the type of germ cultured in sterile biological fluids or identified by ancillary diagnostic examinations.

Bacteremia was defined as the presence of viable bacteria in the circulating blood. Occult bacteremia was defined as bacteremia not associated with clear foci of infection. In this study, single blood culture positive for organisms consistent with skin flora (coagulase-negative Staphylococci, Corynebacterium spp, and alpha-hemolytic Streptococci) was considered contaminated. Respiratory samples were considered positive if a bacterial yield in cultures of valid sputum of at least $10^6$ CFU/ml or a bacterial yield in cultures of bronchoalveolar lavage (BAL) of at least $10^4$ CFU/ml were documented. In this study, Candida spp. isolated from sputum or BAL were considered colonizers unless also present at multiple other sites. Urinary tract infection in men was defined as acute if the symptoms were consistent with the diagnosis and bacteria $\geq 10^4$ CFU/mL were documented on midstream urine specimens. Urinary tract infection in patients with an indwelling catheter was adjudicated by fever >38° C, pain above the pubic bone or in the flank, in addition to one of the following: urine culture with $\geq 10^5$ CFU/mL regardless of the results of the urinalysis, urine culture with $\geq 10^3$ CFU/mL and evidence of pyuria. Specimens were sent for culture only when skin, soft tissue, and bone infections were suspected. Sepsis was considered microbiologically documented only in cases of positive microbiological results derived from deep tissue by biopsy or curettage; otherwise, sepsis was considered clinically documented.

*Source of infection*

When clinical work-up revealed one source of infection, the case was classified as a single source of infection; if at least two unrelated sources of infection were identified, the case was classified as infection with multiple sources. Lower respiratory tract (LRT), urinary tract, skin, soft tissues and bone, and abdominal infections were defined according to guidelines of the Infectious Diseases

Society of America [2-7]. We used the acronym LRTI to identify patients with a single source of infection localized in LRT; the other single sources of infection were grouped as non-LRTI.

*Severity of infection, and definitions of organ dysfunction*

Uncomplicated infection was a SIRS with a definite infective etiology and absence of organ dysfunction. Sepsis was defined as infection associated with signs of hypoperfusion (such as mottled skin, capillary refill ≥ 3 secs, urine output <0.5 ml/kg for at least one hour, or needing dialysis, blood lactate > 18 mg/dl) or organ dysfunction. Organ dysfunction was defined as present when any of the following criteria were met: SaO2< 90%* or $PaO_2/FiO_2$ <300* or ARDS defined as $PaO_2/FiO_2$ <300 and bilateral diffuse opacities, not suggestive of pleural effusion, atelectasis or nodules on bilateral chest X-ray (respiratory dysfunction), increase in serum creatinine of 0.3 mg/dl or ≥ to 1.5 fold from baseline within 48 hours (renal dysfunction), platelets count < 100000/$mm^3$ ** (hematologic dysfunction), apTT >60 sec** or INR> 1.2** or disseminated intravascular coagulation (haemostasis dysfunction), total bilirubin > 4 mg/dl* or INR > 1.5** (liver dysfunction), any form of mental change, inattention, and disorganized thinking indicative of encephalopathy or delirium or any degree of Glasgow Coma Scale worsening (neurologic dysfunction), cardiac arrhythmias*** as high rate atrial fibrillation, high rate narrow complex, ventricular tachycardia, acute coronary syndromes, and acute decompensated heart failure*** (cardiovascular dysfunction). High rate atrial fibrillation or narrow complex tachycardia referred to patients who were clinically or hemodynamically unstable (i.e. myocardial ischemia, pulmonary edema, or hypotension). Septic shock was defined as sepsis plus one or both of the following conditions: mean systemic arterial pressure < 60 mmHg (or <80 mmHg compared to usual pressure rates) despite adequate fluid-resuscitation strategy, and need for dopamine, norepinephrine, or epinephrine, despite the

administration of fluids required to maintain mean arterial pressure > 60 mmHg (or > 80 mmHg if patient has hypertension).

*not known to be chronic

**not known to be chronic or due to medications

*** during the concurrent hospital stay for infection, but not primary diagnosis

**VALIDATION OF "EXTENDED" QUICK SEPSIS-RELATED ORGAN FAILURE ASSESSMENT DERIVED IN GROUP 1 (eqSOFA$_1$)**

Out of 1132 undifferentiated patients with suspected sepsis enrolled in the original study, 30 4 (27%) were excluded from Group 1 due to missing values of clinical variables pertaining t o quick sepsis-related organ failure assessment (qSOFA) and/or serum concentrations of bio markers (C-reactive protein [CRP], lactate, procalcitonin, mid-regional proadrenomedullin [MR -proADM], soluble triggering receptor expressed on myeloid cell-1 [sTREM-1], presepsin, solu ble phospholipase A$_2$ group IIA [sPLA$_2$GIIA], and soluble IL-2 receptor $\alpha$ [sCD25]). In Group 1, the best predictive model to identify patients at low risk of death included gender, age, Charlson Index score, qSOFA score, body temperature <36°C, heart rate >90/minute, and whi te blood cell count >12,000/mm$^3$s (eqSOFA$_1$). Out of 304 undifferentiated patients with suspec ted sepsis excluded from Group 1, 230 (76%) had clinical variables pertaining to eqSOFA$_1$ a vailable: accordingly, the latter patients composed the validation cohort for eqSOFA$_1$ (Figure 1s). We did not find statistically significant difference between the inception (Group 1) and validation cohort except for median Charlson Index, sTREM-1, and presepsin concentrations (higher in the inception than validation cohort, $p$=0.01). For the validation cohort, we compu

ted the predicted probability of event using the linear predictor and the intercept of eqSOFA$_1$ estimated on the inception cohort: lp_per_304=0.043*age+0.261*gender+0.803*qSOFA score+0.127*Charlson Index +1.829*body temperature<36°C + 0.688*white blood cells count >12000/mm$^3$ +0.728*heart rate>90/minute. Pred_prob_304 = EXP(-7.78+lp_per_304)/(1+EXP(-7.78+lp_per_304)). The receiver operating curve (ROC) of eqSOFA$_1$ for the validation cohort is reported in Figure 2s. The area under the ROC curve (AUROC) of eqSOFA$_1$ in the inception (0.79, 95% confidence interval [CI] 0.75-0.83) and validation (0.80; 95% CI 0.728-0.876) cohorts were similarly predictive of 30-day mortality ($p$=0.82).

**VALIDATION OF "EXTENDED" QUICK SEPSIS-RELATED ORGAN FAILURE ASSESSMENT DERIVED IN GROUP 2 INTEGRATED WITH SERUM CONCENTRATIONS OF C-REACTIVE PROTEIN AND MID-REGIONAL PROADRENOMEDULLIN (eqSOFA2+CRP+MR-proADM)**

Out of the 729 patients definitively diagnosed with sepsis at the end of the clinical work-up, 184 (25%) were excluded from Group 2 due to missing value of clinical variables pertaining to qSOFA and/or serum concentrations of biomarkers (CRP, lactate, procalcitonin, MR-proADM, sTREM-1, presepsin, sPLA$_2$GIIA, sCD25, and soluble tumor necrosis factor receptor-1 [sTNFR-1]. In Group 2, the best model to rule out subjects at high risk of mortality encompassed gender, age, Charlson Index score, qSOFA score, body temperature < 36°C, heart rate >90/min, serum concentrations of C-reactive protein, and MR-proADM (eqSOFA$_2$+CRP+MR-proADM). Since all of the aforementioned items were available in 138 of the 184 (75%) patients excluded from Group 2, the former patients were used to compose the validation cohort for eqSOFA$_2$+CRP+MR-proADM (Figure 1s). Only the median concentration of sTREM-1 was significantly higher in the inception (that is Group 2) than in the validation cohort ($p$=0.03). For the validation cohort, we computed the

predicted probability of event using the linear predictor and the intercept of eqSOFA$_2$+CRP+MR-proADM estimated on the inception cohort:

lp_per_184=0.041*age+0.418*gender+0.647*qSOFA+0.03*Charlson Index+0.699*heart rate>90/minute+1.449*body temperature<36°C+0.21*Log C-reactive protein+0.735*Log MR-proADM.

Pred_prob_184 = EXP(-8.58+lp_per_184)/(1+EXP[-8.58+lp_per_184]). The ROC of eqSOFA$_2$+CRP+MR-proADM in the validation cohort is reported in Figure 3s. The AUROC of eqSOFA$_2$+CRP+MR-proADM in the validation cohort (0.82; 95% CI 0.651-0.99) was slightly better than that in the inception cohort (0.80; 95% CI 0.76-0.84; $p$=0.81).

Figure 1s. Flow chart describing the design of the inception (Group 1 and Group 2) and validation cohorts.

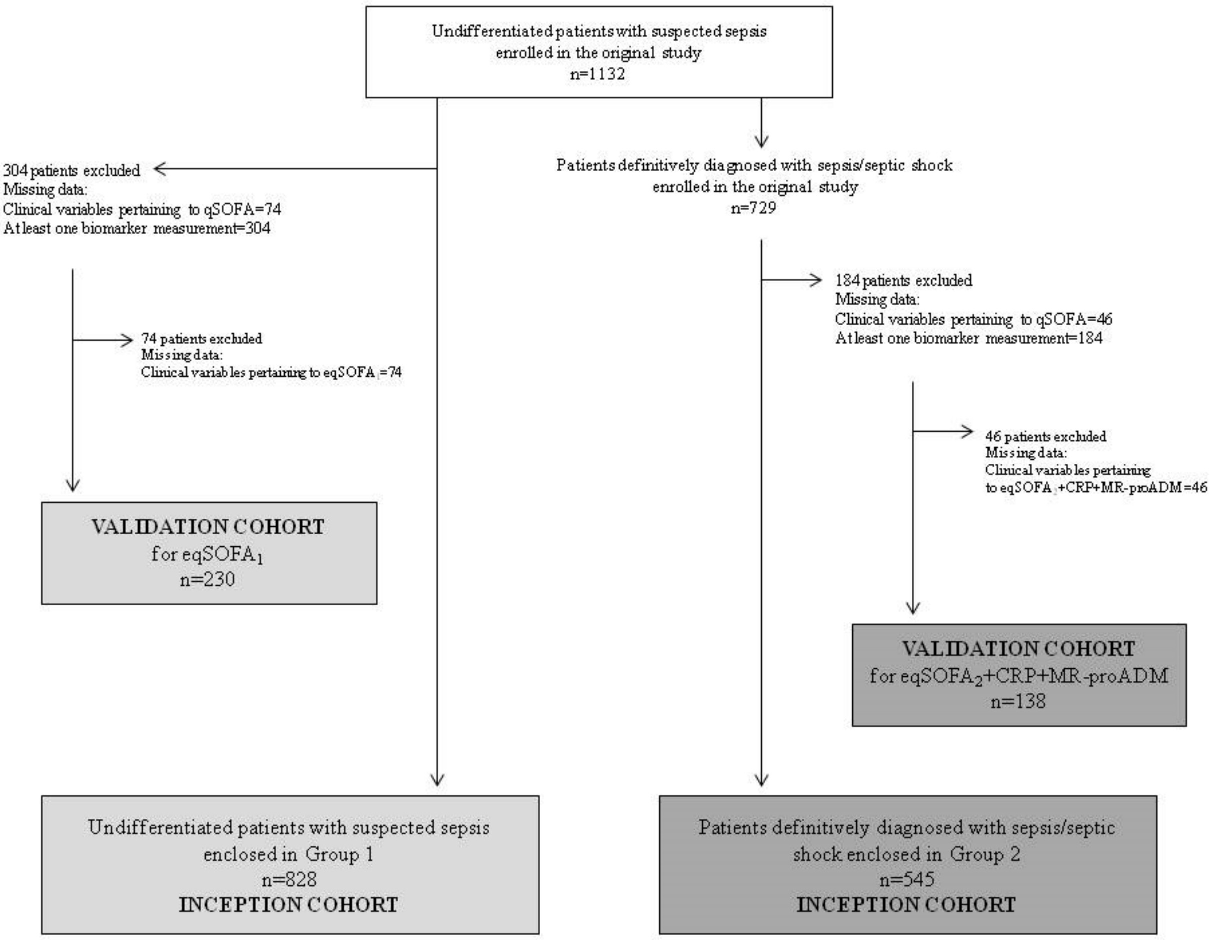


Abbreviations: qSOFA=quick sepsis-related organ failure assessment, eqSOFA$_1$="extended" quick sepsis-related organ failure assessment derived in Group 1 and eqSOFA$_2$+CRP+MR-proADM="extended" quick sepsis-related organ failure assessment derived in Group 2 integrated with serum concentrations of C-reactive protein and mid-regional proadrenomedullin.

Figure 2s. Receiver operating characteristic curve of “extended” quick sepsis-related organ failure assessment derived in Group 1 (eqSOFA1) to predict 30-day mortality in the validation cohort.

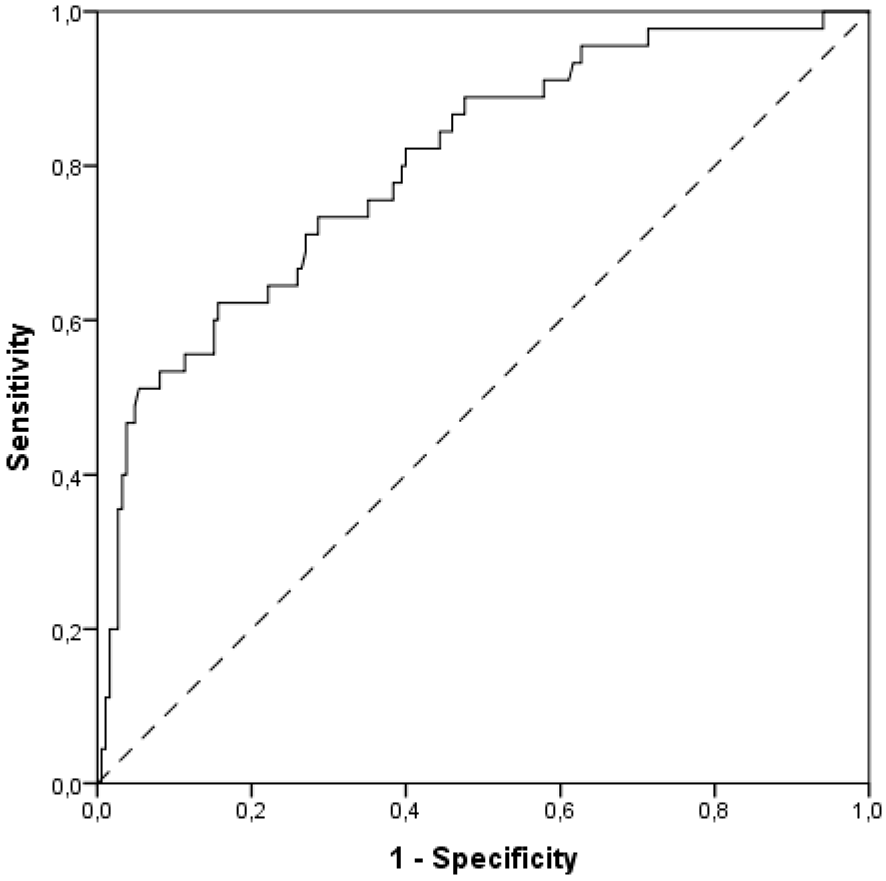

Figure 3s. Receiver operating characteristic curve of the “extended” quick sepsis-related organ failure assessment derived in Group 2 integrated with serum concentrations of C-reactive protein and mid –regional proadrenomedullin (eqSOFA2+CRP+MR-proADM) to predict 30-day mortality in the validation cohort.

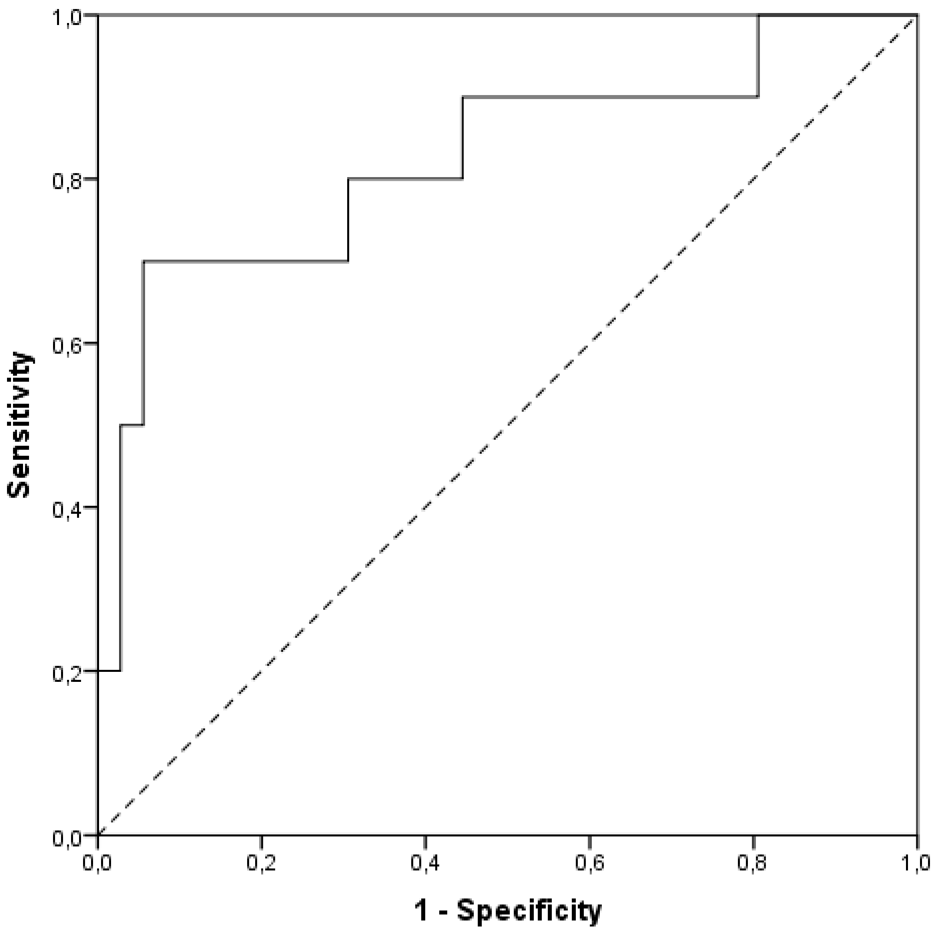

Table 1s. Independent predictors of 30-day mortality in Group 1: multivariable analysis

| **Variables** | **Odds ratio** | **95% confidence interval** | ***p* value** |
|---|---|---|---|
| Gender | 1.51 | 0.98-2.33 | 0.0608 |
| Age | 1.97 | 1.39-2.78 | 0.0001 |
| Charlson Index | 1.30 | 0.99-1.7 | 0.0526 |
| qSOFA | 5.46 | 2.31-12.87 | 0.0001 |
| Body temperature <36°C | 4.36 | 1.77-10.74 | 0.0014 |
| Heart rate>90/min | 1.90 | 1.12-3.24 | 0.0167 |
| White blood cell count >12000/mm$^3$ | 1.52 | 0.96-2.43 | 0.0730 |
| Log C-reactive protein | 1.77 | 1.151-2.74 | 0.0094 |
| Log lactate | 1.49 | 1.11-2 | 0.0069 |
| Log MR-proADM | 1.62 | 1.06-2.47 | 0.0246 |

Abbreviations: qSOFA=quick sepsis-related organ failure assessment, and MR-proADM=mid-regional proadrenomedullin

Table 2s. Independent predictors of 30-day mortality in Group 2: multivariable analysis

| **Variables** | **Odds ratio** | **95% confidence interval** | ***p* value** |
|---|---|---|---|
| Gender | 1.54 | 0.94-2.5 | 0.0823 |
| Age | 1.62 | 1.18-2.23 | 0.0025 |
| Body temperature <36°C | 4.11 | 1.58-10.67 | 0.0036 |
| Heart rate>90/min | 2.03 | 1.13-3.65 | 0.0177 |
| qSOFA | 1.83 | 1.33-2.52 | 0.0002 |
| Log C-reactive protein | 1.65 | 1.06-2.53 | 0.0238 |
| Log MR-proADM | 1.75 | 1.014-3.03 | 0.0444 |

Abbreviations: qSOFA=quick sepsis-related organ failure assessment, and MR-proADM=mid-regional proadrenomedullin.

Table 3s. Prognostic performance of the biomarkers assayed in Group 1.

| | **AUROC** | **Sensitivity** | **Specificity** | **NPV** | **PPV** | **LR-** | **LR+** |
|---|---|---|---|---|---|---|---|
| C-reactive protein | 0.61 (0.57 – 0.66) | 0.72 (0.64 – 0.79) | 0.49 (0.46 – 0.53) | 0.89 (0.85 – 0.90) | 0.24 (0.21 – 0.31) | 0.57 (0.44 – 0.75) | 1.42 (1.25 – 1.61) |
| Lactate | 0.64 (0.60 – 0.69) | 0.74 (0.66 – 0.81) | 0.50 (0.46 – 0.54) | 0.90 (0.86 – 0.91) | 0.24 (0.22 – 0.33) | 0.51 (0.38 – 0.68) | 1.49 (1.32 – 1.69) |
| Procalcitonin | 0.61 (0.57 – 0.66) | 0.68 (0.60 – 0.76) | 0.52 (0.48 – 0.56) | 0.88 (0.84 – 0.90) | 0.24 (0.21 – 0.30) | 0.60 (0.47 – 0.78) | 1.43 (1.24 – 1.63) |
| MR-proADM | 0.74 (0.69 – 0.78) | 0.76 (0.69 – 0.83) | 0.63 (0.59 – 0.66) | 0.92 (0.89 – 0.93) | 0.31 (0.28 – 0.40) | 0.37 (0.28 – 0.50) | 2.06 (1.80 – 2.35) |
| sIL2Rα | 0.65 (0.60 – 0.70) | 0.54 (0.45 – 0.62) | 0.71 (0.68 – 0.74) | 0.87 (0.83 – 0.90) | 0.29 (0.25 – 0.36) | 0.65 (0.55 – 0.78) | 1.85 (1.53 – 2.24) |
| Presepsin | 0.67 (0.63 – 0.71) | 0.78 (0.70 – 0.84) | 0.50 (0.47 – 0.54) | 0.91 (0.87 – 0.92) | 0.25 (0.23 – 0.34) | 0.44 (0.32 – 0.60) | 1.58 (1.40 – 1.77) |
| sTREM-1 | 0.72 (0.67 – 0.76) | 0.74 (0.66 – 0.81) | 0.59 (0.55 – 0.63) | 0.91 (0.88 – 0.92) | 0.28 (0.25 – 0.37) | 0.43 (0.33 – 0.58) | 1.81 (1.59 – 2.06) |
| $sPLA_2GIIA$ | 0.55 (0.50 – 0.60) | 0.70 (0.62 – 0.77) | 0.43 (0.39 – 0.47) | 0.87 (0.82 – 0.88) | 0.21 (0.19 – 0.28) | 0.69 (0.53 – 0.79) | 1.23 (1.09 – 1.39) |

Abbreviations: MR-proADM=mid-regional proadrenomedullin, sIL2Rα=soluble IL-2 receptor α, sTREM-1=soluble triggering receptor expressed on myeloid cell-1, and $sPLA_2GIIA$ = soluble phospholipase $A_2$ group IIA. Cut-off of the biomarkers were selected according to the Youden Index method.

Table 4s. Prognostic performance of the biomarkers assayed in Group 2.

| | **AUROC** | **Sensitivity** | **Specificity** | **NPV** | **PPV** | **LR-** | **LR+** |
|---|---|---|---|---|---|---|---|
| C-reactive protein | 0.58 (0.53 – 0.63) | 0.70 (0.61 – 0.78) | 0.48 (0.43 – 0.53) | 0.84 (0.79 – 0.87) | 0.28 (0.24 – 0.37) | 0.63 (0.47 – 0.84) | 1.33 (1.15 – 1.55) |
| Lactate | 0.59 (0.53 – 0.65) | 0.41 (0.32 – 0.50) | 0.77 (0.73 – 0.81) | 0.82 (0.76 – 0.85) | 0.34 (0.29 – 0.43) | 0.76 (0.65 – 0.89) | 1.82 (1.38 – 2.40) |
| Procalcitonin | 0.57 (0.52 – 0.62) | 0.73 (0.64 – 0.80) | 0.42 (0.37 – 0.47) | 0.84 (0.78 – 0.87) | 0.27 (0.23 – 0.36) | 0.64 (0.47 – 0.88) | 1.26 (1.10 – 1.44) |
| MR-proADM | 0.69 (0.64 – 0.74) | 0.77 (0.68 – 0.84) | 0.55 (0.50 – 0.59) | 0.89 (0.84 – 0.90) | 0.33 (0.29 – 0.44) | 0.42 (0.30 – 0.59) | 1.70 (1.47 – 1.95) |
| sIL2R*α* | 0.61 (0.55 – 0.66) | 0.53 (0.44 – 0.62) | 0.66 (0.61 – 0.70) | 0.83 (0.77 – 0.86) | 0.31 (0.27 – 0.40) | 0.71 (0.58 – 0.87) | 1.56 (1.27 – 1.93) |
| Presepsin | 0.63 (0.57 – 0.68) | 0.77 (0.68 – 0.84) | 0.44 (0.39 – 0.49) | 0.87 (0.81 – 0.89) | 0.28 (0.25 – 0.38) | 0.51 (0.37 – 0.73) | 1.38 (1.21 – 1.57) |
| sTREM-1 | 0.67 (0.62 – 0.72) | 0.73 (0.64 – 0.80) | 0.55 (0.50 – 0.60) | 0.88 (0.82 – 0.89) | 0.32 (0.28 – 0.41) | 0.49 (0.36 – 0.66) | 1.62 (1.40 – 1.89) |
| $sPLA_2GIIA$ | 0.52 (0.46 – 0.58) | 0.76 (0.68 – 0.83) | 0.35 (0.30 – 0.40) | 0.84 (0.77 – 0.86) | 0.25 (0.22 – 0.35) | 0.68 (0.48 – 0.96) | 1.17 (1.04 – 1.32) |
| sTNFR-1 | 0.65 (0.6 – 0.71) | 0.71 (0.62 – 0.79) | 0.55 (0.50 – 0.60) | 0.86 (0.81 – 0.88) | 0.33 (0.28 – 0.43) | 0.52 (0.39 – 0.70) | 1.59 (1.35 – 1.87) |

Abbreviations: MR-proADM=mid-regional proadrenomedullin, sIL2Rα=soluble IL-2 receptor α, sTREM-1=soluble triggering receptor expressed on myeloid cell-1, and $sPLA_2GIIA$ = soluble phospholipase $A_2$ group IIA., and sTNFR-1=, soluble tumor necrosis factor receptor-1. Cut-off of the biomarkers were selected according to the Youden Index method.
